# Radiographic analysis of the sacral-2-alar screw trajectory

**DOI:** 10.1186/s13018-021-02626-9

**Published:** 2021-08-23

**Authors:** Yulin Zhao, Baisheng Yuan, Yijun Han, Binglei Zhang

**Affiliations:** 1 Department of Orthopedics, Qilu Hospital (Qingdao), Cheeloo College of Medicine, Shandong University, No. 758 Hefei Road, Shandong 266035 Qingdao, China; 2 Department of Radiology, Qilu Hospital (Qingdao), Cheeloo College of Medicine, Shandong University, Qingdao, Shandong China

**Keywords:** Second sacrum, Sacral ala, Screw, CT, Analysis

## Abstract

**Purpose:**

To explore the feasibility of sacral-2-alar (S2-alar) screw placement by measuring the length, diameter, and angle of the screw trajectory on computed tomography (CT).

**Methods:**

This study selected 100 Han-nationality adults in northern China with a normal spine and pelvis. CT data were imported into PHILIPS software for reconstructing the 3D digital images. The optimal S2-alar screw trajectory was imitated on CT. Parameters including the length of the screw trajectory, sagittal angle, coronal angle, distance between the entry point and the spinous process, and minimum diameter of the screw trajectory were measured to evaluate the application of S2-alar screws.

**Results:**

In total, 48 males and 52 females were included. The average length of the left screw trajectory was 47.18 ± 3.91 mm. The sagittal angle was 29.06 ± 4.00°. The coronal angle was 13.31 ± 6.95°. The distance between the entry point and the spinous process was 21.0 (3.7) mm. The minimum diameter of the screw trajectory was 17.1 (2.3) mm. The average length of the right screw trajectory was 45.46 ± 4.37 mm. The sagittal angle was 23.33 ± 4.26°. The coronal angle was 14.88 ± 6.84°. The distance between the entry point and the spinous process was 22.8 (2.9) mm. The minimum diameter of the screw trajectory was 16.9 (3.1) mm. In women, the average length of the left screw trajectory was 44.80 ± 3.66 mm. The sagittal angle was 32.14 ± 5.48°. The coronal angle was 16.04 ± 7.74°. The distance between the entry point and the spinous process was 21.8 (2.8) mm. The minimum diameter of the screw trajectory was 17.1 (5) mm. The average length of the right screw trajectory was 44.01 ± 3.72 mm. The sagittal angle was 25.12 ± 5.19. The coronal angle was 16.67 ± 8.34°. The distance between the entry point and the spinous process was 21.6 (2.7) mm. The minimum diameter of the screw trajectory was 17 (4.5) mm. As seen from the data, there were significant differences in the minimum diameter of the screw trajectory in both males and females. In females, there were also significant differences between the left and right sides in the coronal angle. Between males and females, there were statistically significant differences in the length of the screw trajectory. There were no statistically significant differences in the other parameters between males and females.

**Conclusion:**

The optimal screw trajectory of the S2-alar screw can be found on CT. The length and deflection angle of the screw meet the clinical requirements. This method is easy to perform and feasible for clinical application.

## Introduction

Lumbosacral fusion procedures have been widely used in clinical practice. In the treatment of moderate-severe lumbar spondylolisthesis and degenerative scoliosis deformity, standalone sacral-1 (S1) screws have a higher rate of fixation failure. This is because (1) the stress of the distal screw is large; (2) the pedicle of the S1 vertebral arch is wide, while the screw is relatively short; and (3) the vertebra contains cancellous bone with less supporting strength. The clinical application of conventional sacral-2-alar (S2-alar) screws is limited due to their weaker pullout resistance. We found that the S2-alar screw trajectory became longer when the screw was placed on the sides of the sacral wings and had a larger angle in the sagittal plane. Therefore, we put forward the application of S2-alar screws. By increasing the screw angle and deflecting the head of the screw to the outside, the screw will cover a longer distance in the sacrum [1, 2]. Prolongation of the bony screw trajectory correspondingly increases the pullout resistance and, in turn, has the effect of enhanced distal fixation. Here, we measured the clinical data of Chinese S2-alar screws by computed tomographic (CT) scans to provide the basis for clinical application.

## Materials and methods

We randomly screened 100 Han-nationality adults in northern China. They included 48 men and 52 women. The mean age was 46.25 ± 14.95 years. The inclusion criteria were a basically normal spine and pelvis and no obvious scoliosis or kyphosis. The exclusion criteria were obvious radiographic scoliosis, kyphosis, transitional vertebra, or other malformations.

### Radiographic analysis

The spine and pelvis were scanned by CT, and three-dimensional (3D) reconstruction was performed. The power was set at 120 kV and 300 mA. The rotation time was 750 ms. The slice thickness was 1 mm. CT data of lumbar-2 to the pelvis were imported into PHILIPS software (PHILIPS, EBW:V4.5.5.51035) for reconstructing the 3D digital images. The entry point was selected as the midpoint between the lateral border of the S1 and S2 foramens. Directing towards the sacral wing, the screw trajectory with the maximal screw distance in the sacrum was imitated as the optimal trajectory. Through repeated imitation by CT, it was concluded that the maximal bony screw trajectory could be reached with a proper angle in the sagittal and coronal planes. The distance between the entry point and the spinous process was measured to ensure normal installation of the connecting rod. The minimum diameter of the expected screw trajectory was measured to ensure normal installation of the screws.

The measurements were performed by two independent investigators. The data were collected and averaged.

#### Imitation by CT and anatomical measurements of the screw trajectory

**Fig. 1:**
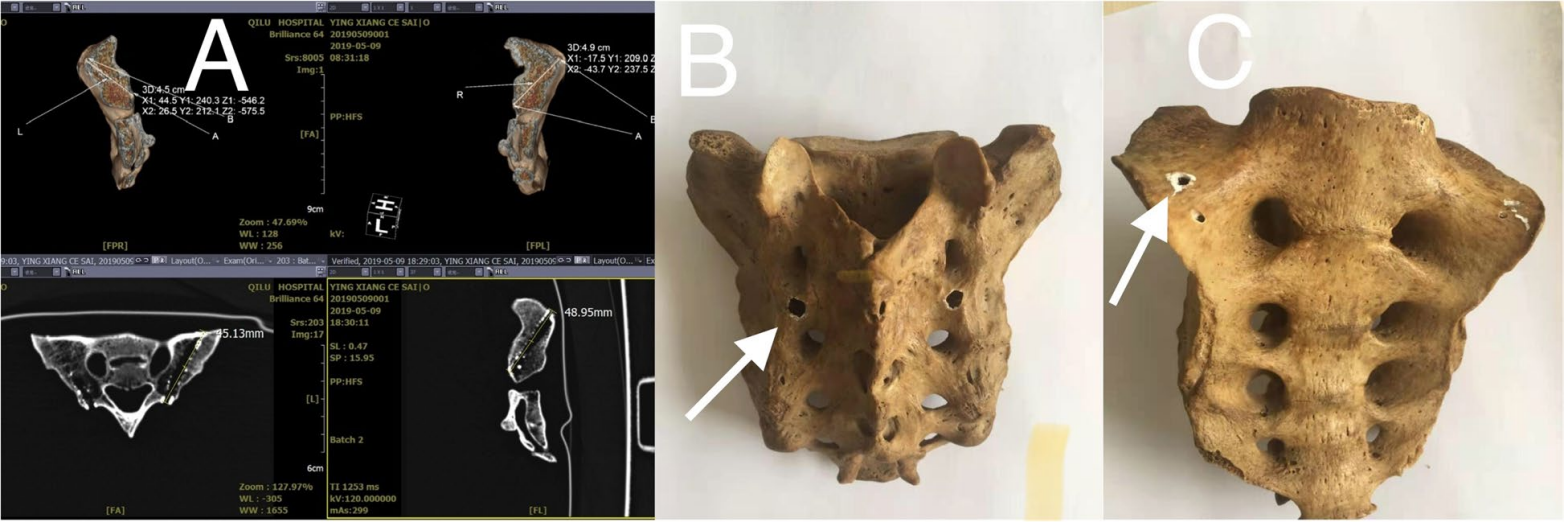
Imitation by CT and anatomical measurements of the screw trajectory

The arrow in Fig. 1B indicates the entry point, and the arrow in Fig. 1C indicates the exit point.

#### The length of the screw trajectory

**Fig. 2:**
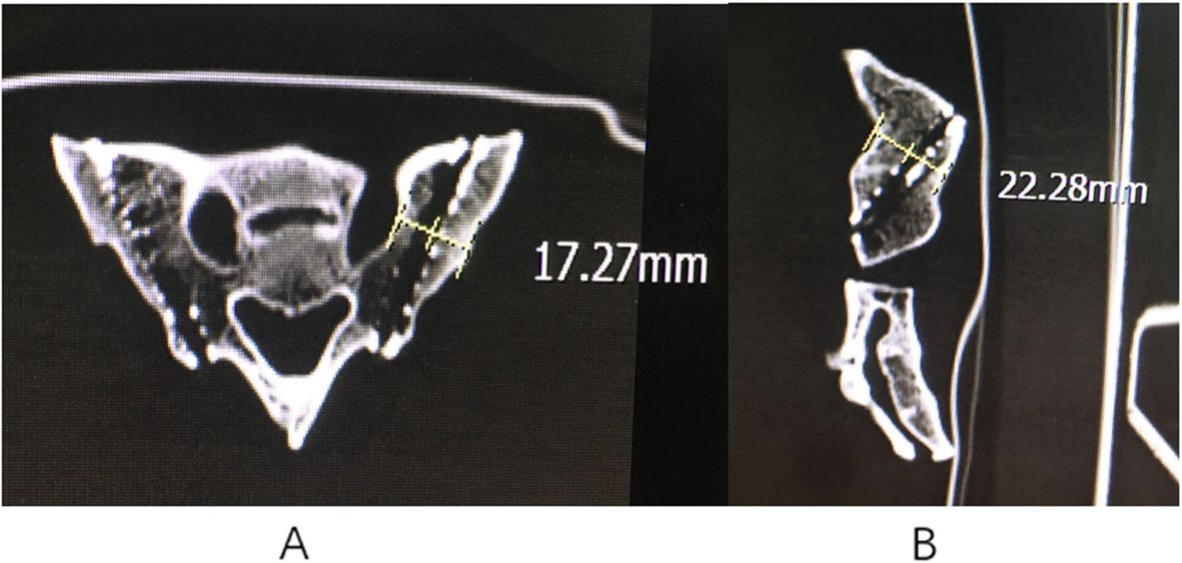
**A** Transverse diameter and **B** sagittal diameter

**Fig. 3:**
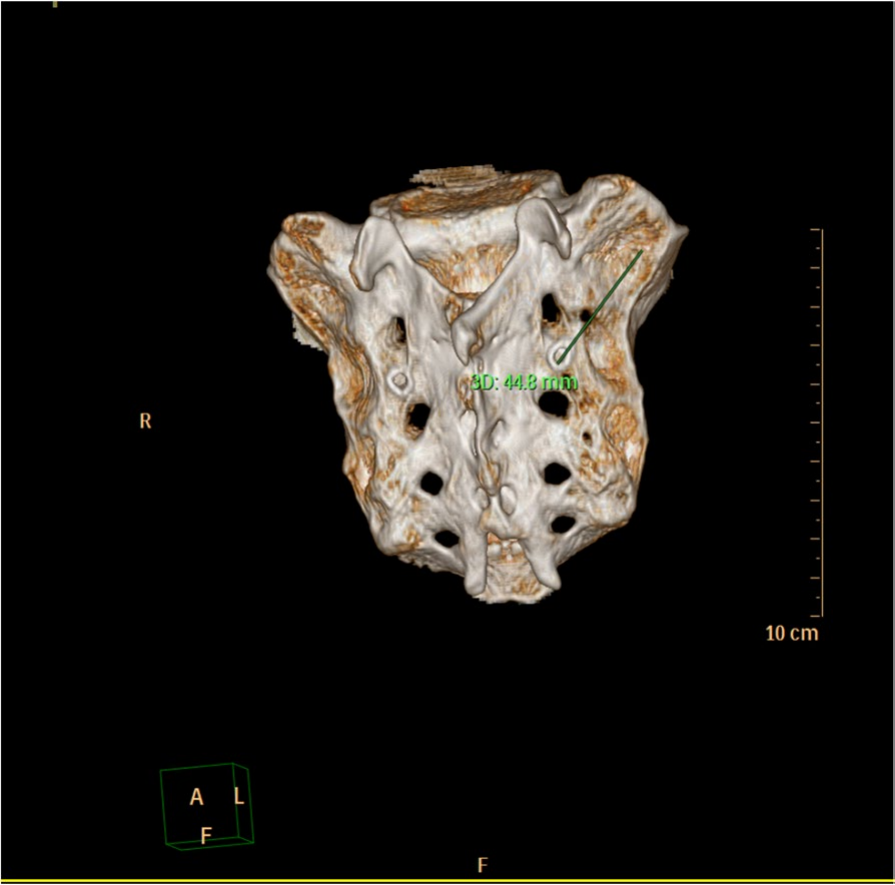
Trajectory length

The transverse diameter and sagittal diameter of the trajectory can meet the requirements of the screw (Fig. 2). The entry point was selected as the midpoint between the lateral border of the S1 and S2 foramens. Directing towards the sacral wing, the screw trajectory with the maximal screw distance in the sacrum was imitated as the optimal trajectory. In this case, the trajectory length reached 45.34 ± 3.96 mm, which provided a sufficient holding force to improve the high complication rate in long-segment distal fixation (Fig. 3).

#### Coronal angle

**Fig. 4:**
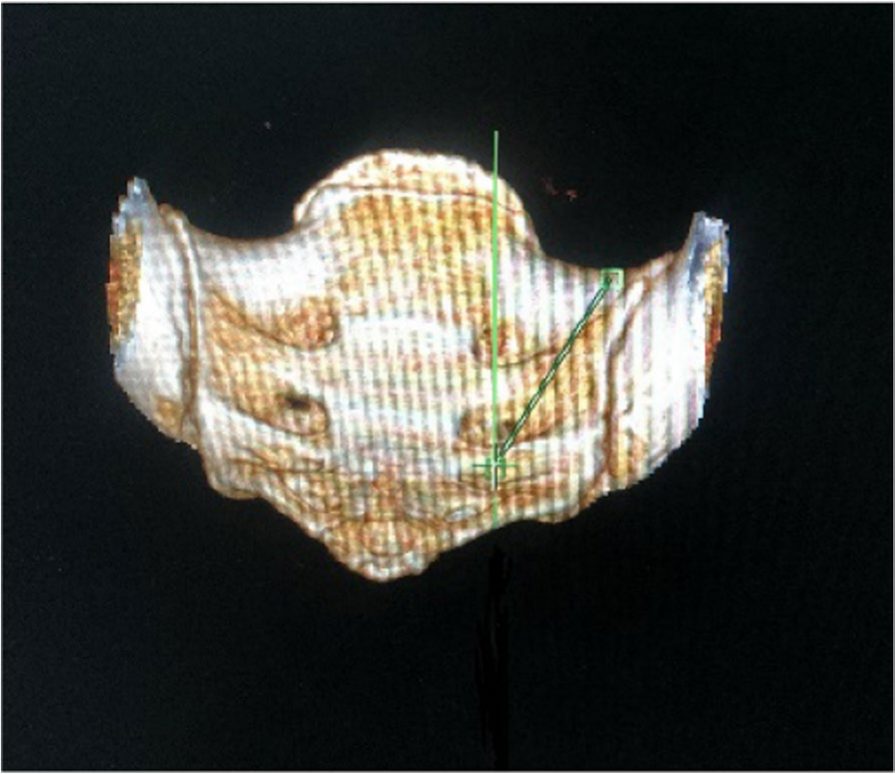
Coronal angle

The caudal trajectory angulation in the coronal plane was measured to meet the needs of screw placement in clinical practice (Fig. 4).

#### Sagittal angle

**Fig. 5:**
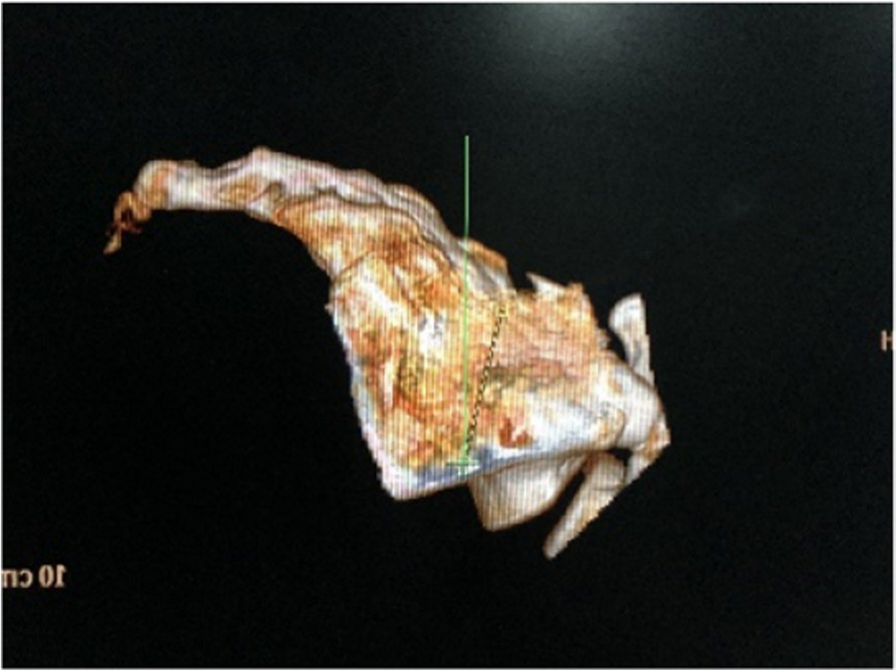
Sagittal angle

The caudal trajectory angulation in the sagittal plane was measured to meet the needs of screw placement in clinical practice (Fig. 5).

#### The distance between the entry point and the spinous process

**Fig. 6:**
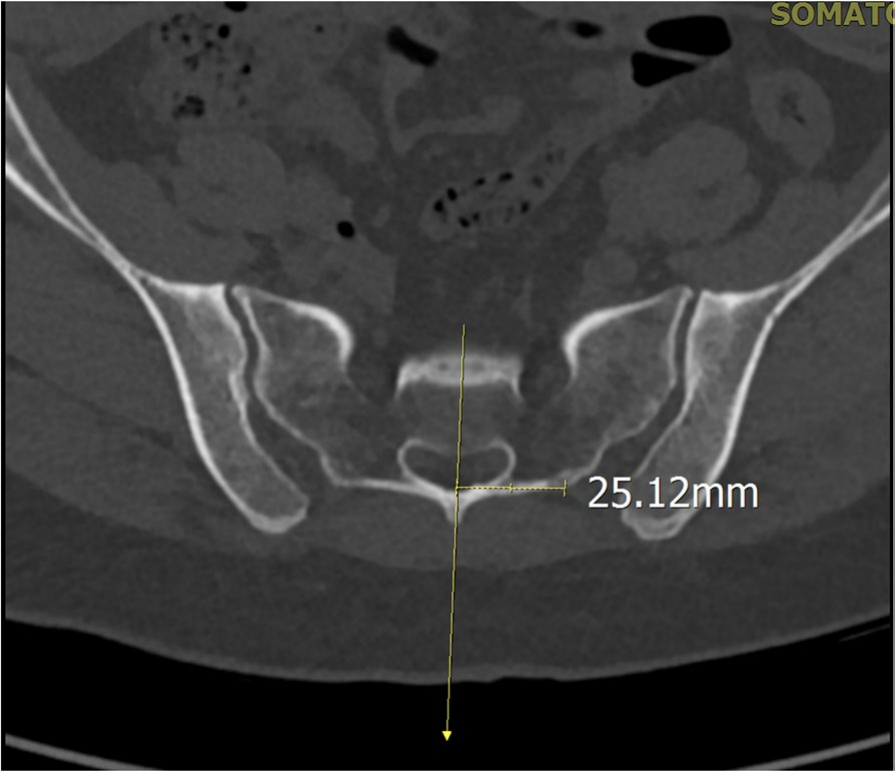
The distance between the entry point and the spinous process

The vertical distance between the entry point and the middle line of the spinous process was measured to verify the accuracy of the entry point and to estimate the convenience of installation of the connecting rod (Fig. 6).

#### Minimum diameter of the screw trajectory

**Fig. 7:**
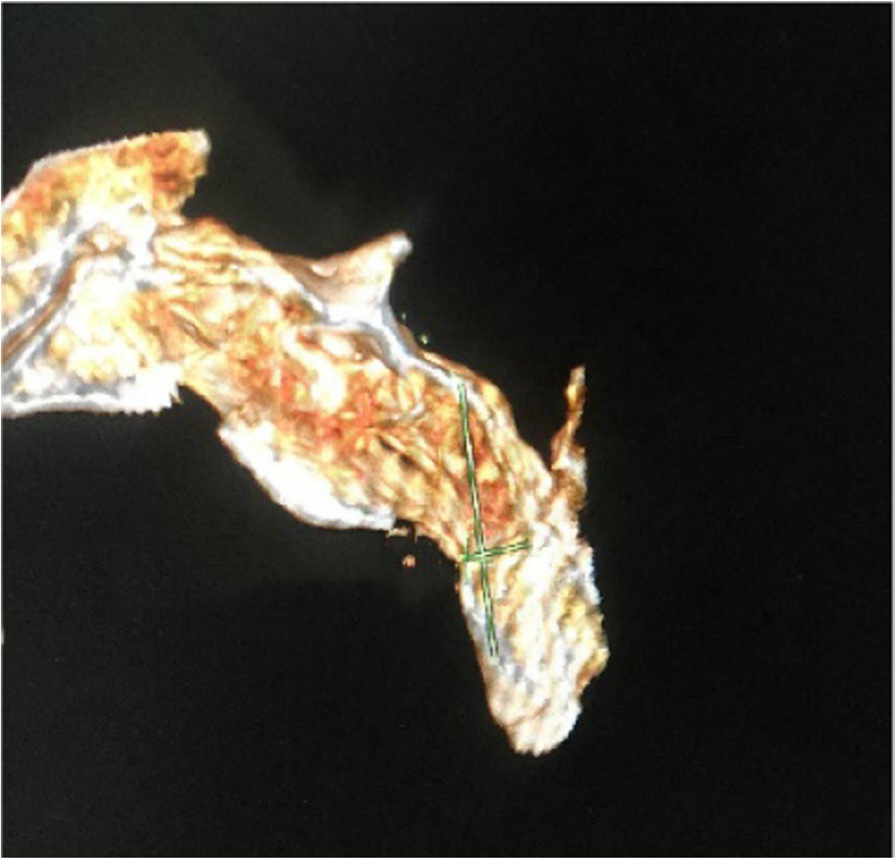
The minimum diameter of the screw trajectory

The minimum diameter of the imitated screw trajectory was measured to ensure safe placement of the screw with a diameter of 4.5–6.5 mm without cutting (Fig. 7).

### Statistical analysis

The Shapiro–Wilk method was used to test the normality of continuous variables. Variables that fit the normal distribution are described as the mean ± standard deviation. Variables that do not fit the normal distribution are described as medians (interquaternary intervals). For the possible differences in parameters between sexes, an independent sample *t* test was used for data conforming to a normal distribution, and the Mann–Whitney *U* test was used for those not conforming to a normal distribution. For the comparison of left- and right-side parameters, the paired *t* test was used for data in line with the normal distribution, while the Wilcoxon signed rank sum test was used for those not in line with the normal distribution. The statistical significance was set at *P* < 0.05. All data were analyzed using SPSS.

## Results

We measured the parameters of 100 normal subjects on CT to obtain S2-alar screw placement data. Forty-eight males and 52 females were included. In males, the average length of the left screw trajectory was 47.18 ± 3.91 mm. The sagittal angle was 29.06 ± 4.00°. The coronal angle was 13.31 ± 6.95°. The distance between the entry point and the spinous process was 21.0 (3.7) mm. The minimum diameter of the screw trajectory was 17.1 (2.3) mm. The average length of the right screw trajectory was 45.46 ± 4.37 mm. The sagittal angle was 23.33 ± 4.26°. The coronal angle was 14.88 ± 6.84°. The distance between the entry point and the spinous process was 22.8 (2.9) mm. The minimum diameter of the screw trajectory was 16.9 (3.1) mm. In women, the average length of the left screw trajectory was 44.80 ± 3.66 mm. The sagittal angle was 32.14 ± 5.48°. The coronal angle was 16.04 ± 7.74°. The distance between the entry point and the spinous process was 21.8 (2.8) mm. The minimum diameter of the screw trajectory was 17.1 (5) mm. The average length of the right screw trajectory was 44.01 ± 3.72 mm. The sagittal angle was 25.12 ± 5.19. The coronal angle was 16.67 ± 8.34°. The distance between the entry point and the spinous process was 21.6 (2.7) mm. The minimum diameter of the screw trajectory was 17 (4.5) mm (Tables 1 and 2).

**Table 1:** Comparison of individual parameters in males and females

	Male (*n* = 48)			Female (*n* = 52)		
	Left	Right	*P*	Left	Right	*P*
Length of the screw trajectory (mm)	47.18 ± 3.91	45.46 ± 4.37	< 0.01	44.80 ± 3.66	44.01 ± 3.72	0.024
Sagittal angle (°)	29.06 ± 4.00	23.33 ± 4.26	< 0.01	32.14 ± 5.48	25.12 ± 5.19	< 0.01
Coronal angle (°)	13.31 ± 6.95	14.88 ± 6.84	0.014	16.04 ± 7.74	16.67 ± 8.34	0.337
Distance between the entry point and the spinous process (mm)	21.0 (3.7)^a^	22.8 (2.9)^a^	0.001^b^	21.8 (2.8)^a^	21.6 (2.7)^a^	0.004^b^
Minimum diameter of the screw trajectory (mm)	17.1 (2.3)^a^	16.9(3.1)	0.638^b^	17.1 (5)^a^	17(4.5)^a^	0.059^b^

The Shapiro–Wilk test showed that the length of the nail track, the angle of the sagittal plane, and the angle of the coronal plane were all normally distributed (*P* > 0.05); the distance between the nail entry point and the spinous process and the minimum diameter of the nail path had a non-normal distribution

^a^the median (quartile spacing), ^b^the Wilcoxon sign rank sum test

**Table 2:** Comparison of parameters between males and females

	Male (*n* = 48)	Female (*n* = 52)	*P*
Length of the screw trajectory (left) (mm)	47.18 ± 3.91	44.80 ± 3.66	0.002
Length of the screw trajectory (right) (mm)	45.46 ± 4.37	44.01 ± 3.72	0.075
Sagittal angle (left) (°)	29.06 ± 4.00	32.14 ± 5.48	0.002
Sagittal angle (right) (°)	23.33 ± 4.26	25.12 ± 5.19	0.065
Coronal angle (left) (°)	13.31 ± 6.95	16.04 ± 7.74	0.068
Coronal angle (right) (°)	14.88 ± 6.84	16.67 ± 8.34	0.244
Distance between the entry point and the spinous process (left) (mm)	21.0 (3.7)^a^	21.8 (2.8)^a^	0.423^b^
Distance between the entry point and the spinous process (right) (mm)	22.8 (2.9)^a^	21.6 (2.7)^a^	0.143^b^
Minimum diameter of the screw trajectory (left) (mm)	17.1 (2.3)^a^	17.1 (5)^a^	0.492^b^
Minimum diameter of the screw trajectory (right) (mm)	16.82 ± 2.66	16.54 ± 3.55	0.656

The Shapiro–Wilk test showed that the length of the nail track, the angle of the sagittal plane, the angle of the coronal plane, and the right side of the minimum diameter of the nail track were normally distributed (*P* > 0.05); the distance between the nail entry point and the spinous process and the minimum diameter of the nail path had a non-normal distribution

^a^the median (interquartile interval), ^b^the Mann–Whitney *U* test

As seen from the data, there were significant differences in the minimum diameter of the screw trajectory between males and females. In females, there were also significant differences between the left and right sides in the coronal angle. Between males and females, there were statistically significant differences in the length of the screw trajectory. There were no statistically significant differences in the other parameters between males and females.

The results showed that the S2-alar screw had a sufficient screw trajectory length. The entry point of the screw had obvious anatomical marks, and the distance between the entry point and the spinous process was relatively fixed. The screw placement was operable and repeatable.

## Discussion

Spinal malformation is common in clinical spinal surgery and is also one of the important reasons for surgical treatments of spinal diseases. Among these conditions, lumbar spondylolisthesis and degenerative scoliosis of the lumbar spine often require surgical correction and internal fixation. Severe cases often need fixation to the sacrum or even the pelvis to achieve the requirements of correction and stability. Complications such as loosening of internal fixation, broken screws, pseudarthrosis, and pain are often encountered after traditional spinal and pelvic fixation techniques [3, 4]. The use of iliac screws and trans-S2 sacroiliac screws has significantly reduced the complications associated with internal fixation. However, related clinical problems, such as sacroiliac joint degeneration and sacroiliac arthritis, have also been reported [5]. Neurovascular injury involving screw trajectory deviation of the iliac bone and sacroiliac joint is also one of the problems that needs to be given great attention [6].

Spinal pelvis fixation remains a challenging area in spine surgery for the treatment of moderate-to-severe spondylolisthesis and degenerative scoliosis and involves biomechanical balance problems, deterioration of physiological functions, and revision of internal fixation with a high failure rate. For severe degenerative scoliosis of the lumbar spine, severe spondylolisthesis, revision surgery, etc., long-segment fixation with distal fixation to the pelvis is often required. Distal fixation is of critical importance in thoracolumbar malformation surgery. Multiple studies [7] have shown that when S1 pedicle screws are used without auxiliary fixation, the incidence of pseudarthrosis is higher and this approach is associated with fixation failure and poor prognosis [8].

Due to the high failure rate of S1 screws, iliac screws and S2 ala-iliac (S2AI) screws were developed. The latter two have high reliability of internal fixation due to tricortical fixation. S2AI screws are especially popular because of their low notch and firm fixation. Both iliac screws and S2AI screws force the sacroiliac joint or directly fix the sacroiliac joint. Degeneration and fusion of the sacroiliac joint, as well as pain, are potential risks. S2-alar screws have been clinically applied since the 1980s and have received attention from and been researched by clinical peers. However, due to the short bony screw trajectory and insufficient holding force, the clinical application of traditional S2-alar screws must be abandoned. The development of sacral screws, from the initial Galveston iliac rod, Jackson sacral rod, and Kostuik trans-iliac rod to the later spine-iliac screw and trans-S2 sacroiliac screw [9–11], has resulted in a significant reduction in trans-structural complications. The iliac screw, which connects the spine to the pelvis across the plane, greatly increases the stability of fixation [12]. Complications, including internal fixation fracture, delayed bone healing, pain, and skin ulceration, have also occurred with clinical use. In recent years, S2AI screws have been formed through exploration of the trajectory of trans-S2 sacroiliac screws. Numerous studies have demonstrated the effectiveness and stability of S2AI screws, as well as fewer pseudarthrosis formations and fewer internal fixation failures [13–17]. At the same time, some scholars have shown that screw fracture also occurs in the process of bone healing due to the micro-mobility of the sacroiliac joint [18]. In addition, the degeneration and pain of the sacroiliac joint might require further study. In terms of technical difficulty, S2AI requires more skilled screw placement techniques and better surgeons. This difficulty can be reduced through the application of intraoperative navigation technology but likely poses certain challenges to hospitals that lack this technology [19, 20]. At present, S2AI has been established as the preferred fixation mode for the spine and pelvis. S2AI can provide relatively strong internal fixation and greatly reduce the problem of distal fixation failure. However, there are still new clinical problems in the clinical application of S2AI. Distal fixation across the joint to pelvic fixation may lead to new clinical problems. S2AI can reduce the incidence of complications mainly because of its stable distal fixation, which can achieve perfect distal fixation in the bone-healing stage [21].

In this paper, the radiographic measurements of S2-alar screws were described.

The entry point was selected as the midpoint between the lateral border of the S1 and S2 foramens. Directing towards the anterolateral of the sacral wing, because the anterolateral sacral wing has plenty of bone and ample space, the S2-alar screw did not pass through the sacroiliac joint. Its length and biomechanics might meet the requirements of screw placement. In this way, it could avoid stress or damage to the sacroiliac joint in the distal fixation at long segments and might effectively prevent or reduce later pain or degeneration of the sacroiliac joint and decrease postoperative complications. Our results showed that by adjusting the screw trajectory and deflecting the fixed S2 screw, the length of the screw trajectory reached 45.34 ± 3.96 mm so that there was enough holding force to improve the drawbacks of traditional S2 screws. S2-alar screw placement is relatively simple, safe, and stable.

The sacrum is an irregular bone, and the S2-alar screw is a new screw placement method. 3D printing is also a method to improve the patient-doctor relationship and to avoid liabilities. Explaining the surgery to the patient using 3D models could improve his preoperative emotional status and could also help him overcome the fear of surgery.

Such models could also be used in defensive medical decision-making [22].

This study is only a preliminary work for the design and improvement of S2-alar screws. The results confirmed the feasibility of S2-alar screw placement. In the next step, we will conduct further finite element analysis and biomechanical experiments to confirm the reliability and stability of the improved S2-alar screw.

## Conclusions

Through the above-described measurements, we found that there were few individual differences in the parameters of S2-alar screw placement. Although there were significant differences in the minimum diameter and length of the screw trajectory between males and females, all individuals could obtain the optimal screw trajectory. Based on the previous data, we proposed a scheme of S2-alar screw placement. By designing the screw trajectory and measuring the parameters, it was proven that the S2-alar screw could be applied to most people, which provides the radiographic basis for further verification of its clinical effectiveness.

## Data Availability

All data generated or analyzed during this study are included in this published article.

## Abbreviations

CT: Computed tomography
S2-alar: Sacral-2-alar
3D: Three-dimensional
S2AI: S2 ala-iliac
